# Synthesis and Structural Characterization of CaO-P_2_O_5_-CaF:CuO Glasses with Antitumoral Effect on Skin Cancer Cells

**DOI:** 10.3390/ma15041526

**Published:** 2022-02-18

**Authors:** Nicoleta Simona Vedeanu, Cristian Lujerdean, Marius Zăhan, Daniel Severus Dezmirean, Lucian Barbu-Tudoran, Grigore Damian, Răzvan Ștefan

**Affiliations:** 1 Department of Pharmaceutical Physics-Biophysics, Faculty of Pharmacy, “Iuliu Hatieganu” University of Medicine and Pharmacy, Pasteur 6, 400349 Cluj-Napoca, Romania; simona.vedeanu@umfcluj.ro; 2 Faculty of Animal Science and Biotechnology, University of Animal Sciences and Veterinary Medicine Cluj-Napoca, 400372 Cluj-Napoca, Romania; mzahan@usamvcluj.ro (M.Z.); ddezmirean@usamvcluj.ro (D.S.D.); 3 Electron Microscopy Center “Prof. C. Craciun”, Faculty of Biology & Geology, “Babes-Bolyai” University, 5-7 Clinicilor St., 400006 Cluj-Napoca, Romania; lucian.barbu@ubbcluj.ro; 4 National Institute for Research and Development of Isotopic and Molecular Technologies of Cluj-Napoca, 400293 Cluj-Napoca, Romania; 5 Faculty of Physics, Babes-Bolyai University, 400084 Cluj-Napoca, Romania; grigore.damian@ubbcluj.ro

**Keywords:** phosphate glass, dissolution tests, copper ions, Fourier transform infrared (FTIR) spectra, antitumor activity, MTT assay

## Abstract

Copper is one of the most used therapeutic metallic elements in biomedicine, ranging from antibacterial approaches to developing new complexes in cancer therapy. In the present investigation, we developed a novel xCuO∙(100 − x) [CaF_2_∙3P_2_O_5_∙CaO] glass system with 0 ≤ x ≤ 16 mol% in order to determine the influence of doping on the composition structure of glasses. The samples were characterized by dissolution tests, pH measurements, Fourier-transform infrared spectroscopy (FT-IR), electron paramagnetic resonance (EPR), Scanning Electron Microscopy with energy dispersive spectroscopy (SEM-EDX) and afterward, their antitumor character was assessed. The glasses were mostly soluble in the aqueous medium, their dissolution rate being directly proportional to the increase in pH and the level of doping up to x = 8 mol%. FT-IR spectra of glass samples show the presence of all structural units characteristic to P_2_O_5_ in different rates and directly depending on the depolymerization process. SEM-EDX results revealed the presence of an amorphous glass structure composed of P, O, Ca, and Cu elements. The 3-(4,5-dimethylthiazol-2-yl)-2,5-diphenyltetrazolium bromide (MTT) reduction assay showed strong cytotoxicity for tumoral cells A375 even in low concentrations for Cu-treatment. In contrast, the copper-free matrix (without Cu) determined a proliferative effect of over 70% viability for all concentrations used.

## 1. Introduction

The skin is a vital multifunctional organ; it covers the entire body surface and it plays an essential role in regulating temperature, as well as in protection, perception, absorption, excretion, metabolism, and immunity [1,2]. The structural integrity of the skin is also crucial for maintaining physiological functions. However, in addition to usual skin damage (physical or chemical), skin cancers are occupying a leading place of malignancy in humans, particularly in the Caucasian population in the elderly [3,4,5]. The most common skin cancer types are basal cell carcinomas (BCCs), squamous cell carcinomas (SCCs) (both also referred as nonmelanocytic skin cancer—NMSC), and cutaneous malignant melanomas (CMs) (also designed as malignant melanoma of the skin or melanoma) [6]. The single treatment that exists is conventional surgical excision with an intraoperative histological and margin assessment or Mohs micrographic surgery (MMS), which are considered the gold standard intervention [7]. However, the postoperative tissue healing of tumor excision, persistent inflammation, bacterial infections, and recurrence after a tumor resection can cause big problems in cancer therapy and post-surgical skin regeneration. 

The research in the field of biomedicine, tissue engineering, and biotechnology is in a continuous search for synthetic materials, but also for new remedies or solutions applicable to the regeneration of affected tissue. After decades of research, bioactive glasses and glass-ceramics have become some of the most intensively studied materials due to their revolutionary potential for many health applications, including cancer treatment [8].

Phosphate glass is a favorite material in the field of biomaterials, first because it has the chemical ability to dissolve elements and oxides that are insoluble for other glass materials and second because of its similarities in composition to bones and teeth [9,10]. The chemical addition of various amounts of calcium, sodium, iron, vanadium, copper or barium oxides in the structure of vitreous materials reduced their high hygroscopic degree and increased their durability, making them suitable as biomaterials [11,12,13,14,15,16]. Doping the phosphate material with such metallic ions not only increases chemical durability but also its bioactivity for proper concentrations [17]. Calcium phosphate-based materials are nowadays used by the human body to build bones or to auto-produce material for bone repair and regeneration. They are osteoconductive and some others are also osteoinductive [18]. The insertion of metallic ions in the phosphorous network produces the shift in the position of the band of the ionic groups [13,19,20,21,22,23,24], (PO_4_)^3-^ and the increase of its relative area at the expense of that of P = O and P-O-P; this may be considered as an indication for the formation of more non-bridging oxygen ions (NBOs). According to Karlsson and Ylanen statements [17], this fact is related with a potential bioactivity. Thus, metallic insertion in small and controlled quantities may have a beneficial role at material level from a biological point of view. Moreover, the importance of calcium for bones is well known. Calcium is not only part of the structure of bones, but it is responsible for the the development and maintenance of healthy bones for both women and men. As for copper [19,20,21,24,25] it is a bio-element necessity for the human body that occurs in calcium and phosphorus metabolism. It exhibits two different ionic oxidation states (Cu (II) or Cu (I)) [26], and copper-containing complexes have been identified as the best candidates for anticancer agents [27,28,29,30,31]. The mechanism underlying the anti-tumor activity of these Cu-based complexes is the increase of ROS production and oxidative stress which causes DNA damage, increased death receptor expression and ultimately apoptosis-mediated cell death. In this regard, Cu2+ ions in such complexes are reduced to Cu+ ions and hydrolytic cleavage occurs in the hypoxic environment of cancer cells [32,33]. 

The present research reports the synthesis of a novel CaO-P_2_O_5_-CaF:CuO bioglass system by doping small amounts of copper and exploring the structural and biological properties regarding their antitumor activity. In this sense, multiple analyses of glasses are made, such as dissolution tests, bioactivity tests (pH measurement), Fourier-transform infrared spectroscopy (FTIR), Scanning Electron Microscopy (SEM) with energy dispersive spectroscopy (EDX), electron paramagnetic resonance (EPR), and the evaluation of the antitumor character (MTT assay) on human melanoma cell line A375.

## 2. Materials and Methods

### 2.1. Materials

Calcium fluoride (CaF_2_, 99.5%) and phosphorus pentoxide (P_2_O_5_, 98%) were purchased from Alfa Aesar (Karlsruhe, Germany). Copper oxide extra pure (CuO, 99.5%) and calcium carbonate pure (CaCO_3_) were purchased from Merck (Darmstadt, Germany) and Petr Švec—PENTA, (Prague, Czech Republic) respectively. For cytotoxicity assay, A375 (CRL-6475™) tumor epithelial cells were used and obtained from American Type Culture (ATCC^®^) (Manassas, VA, USA). All dissolution/extracts were performed with deionized water.

### 2.2. Preparation of Bioactive Glasses

The glass samples were synthesized by the melt-quenching technique [13]. Appropriate chemical compositions of the batches were weighed, mixed in appropriate stoichiometric proportions and ground thoroughly using mortar and pestle to obtain a homogenized composition for each sample (Table 1). Then, the batches were taken into sintered corundum crucibles and melted at 1200 °C in an electrical furnace for 15 min. Homogenized melts were quenched at room temperature by pouring them on a stainless-steel plate and quickly pressing them. 

The prepared glass samples were crushed to a fine powder form for further characterization of the samples. The glasses were mechanically (crushed) and chemically (dissolved) processed according to the analysis package used. Thus, for the experiments in this paper, the following samples were used: (i) pieces of glasses, (ii) powder glass and (iii) glass extracts in the form of colloidal solutions.

### 2.3. Structural Characterization of Bioactive Glasses

#### 2.3.1. Dissolution Tests

The solubility of powdered glasses was determined by measuring their weight loss after immersion of 100 mg microparticle glass in 10 mL of deionized water for 14 days at 37 °C using a Nüve EN-025 incubator. After fourteen days of immersion, the resulting suspensions were centrifuged, decanted and vacuum-filtered by using Whatmann 41 filter paper and the resulting sediment was dried at room temperature for 72 h. After drying, it was weighed using a Precise XT220A analytical balance with a self-calibration system (SCS), where the relative weight loss (*W_loss_*) of each vitreous sample was expressed as a percentage according to the equation:

(1) $$W_{loss}=\frac{m_{i}-m_{f}}{\;m_{i}}\times 100$$

where *m_i_* is the initial weight of each sample (*m_i_* ≅ 100 mg powdered glass sample) and *m_f_* is the final weight after 14 days of immersion in deionized water.

Furthermore, the percentage of mass loss for this system was measured also by using pieces of glass for an immersion time of 696 h (29 days) at 37 °C. Glass samples (pieces) close in size and weight were chosen for these measurements. Briefly, these vitreous oxide samples were weighed before immersion (*m_i_*) and after each immersion time (*m_f_*), respectively (0 h, 48 h, 144 h, 216 h, 408 h and 696 h). Immediately after incubation, they were wiped off to remove excess water, dried for 4 h at 60 °C and re-weighed.

#### 2.3.2. pH Evolution

The in vitro experiment was performed by soaking the glass samples in phosphate-buffered saline (PBS) for 21 days. It was synthesized according to AAT Bioquest, Inc. [34] and were used the reagent grade chemicals NaCl, KCl, Na_2_HPO_4_ and KH_2_PO_4_ were dissolved one by one into distilled water and the final solution is buffered to pH 7.4 (Table 2). The powder samples were soaked in PBS and stirred every 3 days for 21 days at 37 °C. The ratio of powder to solution was 10:1 (mg/mL). The pH measurements were made at different incubation periods (0, 1, 2, 5, 9, 14 and 21 days) with the purpose to observe its evolution.

#### 2.3.3. FT-IR

Fourier transform infrared spectroscopy (FT-IR) was performed in order to identify the correlation between absorption wavelengths and the chemical structure of the samples. The analysis was performed on powder samples for xCuO (100 − x)(CaF_2_∙3P_2_O_5_∙CaO) glass system with 0 ≤ x ≤ 16 mol%). In this regard, 0.005 g of vitreous oxide powders were homogenized with 0.2 g of potassium bromide (KBr), which were subsequently pressed in the form of pellets with a Manual Hydraulic Press (Specac Ltd, Orpington, UK) for 3 min/10 tons. The spectra were recorded using the Jasco FT-IR 4100 spectrometer with a spectral resolution of 4 cm^−1^, in the range of 350–4000 cm^−1^ wave numbers, with 256 scans/sample.

#### 2.3.4. EPR

The EPR measurements of powder samples were carried at room temperature with Bruker Biospin EMX spectrometer operating at X-band (9–10 GHz). The EPR parameters were set at 100 KHz modulation frequency, microwave power 10 mW, modulation amplitude 3 G; time constant 2.56 ms; scan time 61 s; receiver gain 10^3^. To avoid the alteration of the glass structure due to the ambient conditions, especially humidity, samples were powdered and enclosed in tubular holders of the same caliber. Equal quantities of samples were studied.

#### 2.3.5. SEM-EDX

The morphology and composition of the samples were investigated by analyzing the surfaces with an electron microscope (SEM) and the distribution of the contained elements by X-ray scattering spectroscopy (EDX), respectively. SEM and EDX analyzes were recorded using a high-resolution scanning electron microscope equipped with a Hitachi SU8230 cold-field emission gun (Japan), and to obtain high-quality images, the glass samples were metallized with a thin gold (10 nm) layer, using a Polaron E-5100 plasma spray gun (Polaron Equipment Ltd., Watford, UK), in the presence of Argon (45 s at 2 kV and 20 mA). For a better interpretation, the obtained signals were collected from at least two different areas with different surface magnifications, averaging the measured concentrations. Moreover, the data were processed with special Aztec analysis software programs. Note that all quantitative EDX elementary data presented are semi-quantitative weight percent (% wt) and normalized to 100%.

### 2.4. Assessment of In Vitro Bioactivity Test

#### 2.4.1. Cell Culture

The A375 (ATCC^®^ CRL-1619™) human melanoma cell line was obtained from American Type Culture Collection (Rockville, MD, USA). Tumoral cells were grown in DMEM (Dulbecco’s modified Eagle’s medium) containing 4.5 g/L glucose, supplemented with 10% FBS, 2 mM glutamine, without antibiotics. Cells were cultured in a 5% CO_2_ atmosphere at 37 °C. The culture medium was changed every 2–3 days.

#### 2.4.2. Cytotoxicity Function In Vitro—MTT Assay 

The effects of glasses C6, chosen because its maximum solubility and M (*Matrix*) chosen as a standard, on the viability of tumor cells was evaluated by MTT cell proliferation reagent (Thiazolyl Blue Tetrazolium Bromide, Sigma-Aldrich, St. Louis, MO, USA). Tumor cells were plated (5 × 103 cells/well) in a 96-well plate and exposed to glass extracts for 24 h at temperature 37 °C and 5% CO_2_. 

Five serial concentrations (2, 4, 6, 8, and 10 CuO μg/mL) of C6 and matrix glasses (with the same amounts expressed in equivalent volumes as for sample C6) were tested over tumor cells. The optical density (OD) was measured at 550 and 630 nm (for background) using Synergy HT Multi-Mode Microplate Reader BioTek, USA. The data were presented as the percentage of viable cells calculated from the following Equation (2).

% Cell Viability = [(OD_s_ − OD_b_)/(OD_c_ − OD_b_)] × 100 (2)

where ODs—the optical density (in units) for the sample, OD_b_—the optical density for the blank wells, and OD_c_—the optical density for the control wells. The results were expressed as percent survival relative to an untreated control (CTRL-) and the experiment was performed with five repetitions for each concentration of treatment, for a better and more accurate prediction.

## 3. Results and Discussions

### 3.1. Structural Characterization 

#### 3.1.1. Dissolution Tests

**Figure 1:**
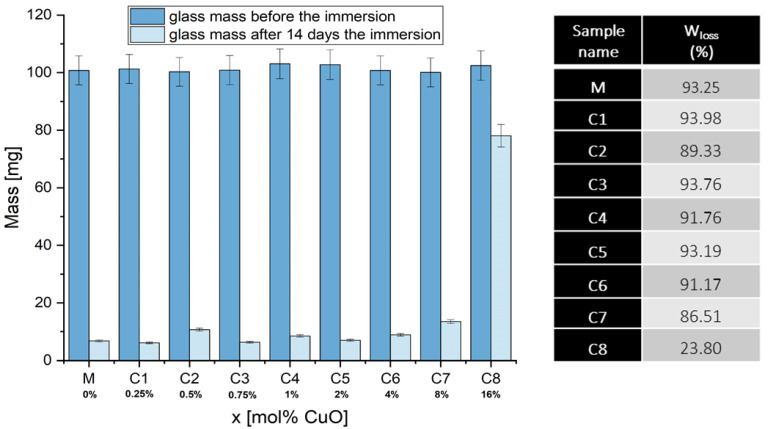
The solubility of powder glass before and after 14 days immersion in 10 mL deionized water.

The solubility test shows that the dissolution rate of glass powders for xCuO ∙ (100 − x) [CaF_2_∙3P_2_O_5_∙CaO] glass system, where x 0 ≤ x ≤ 16 mol% is grown for all samples in the aqueous medium, except the C8 sample (x =16 mol%) (Figure 1). The results suggest that the addition of CuO ions up to x ≤ 8% mol% does not change the solubility of the glasses (C1-C7), as they are as sensitive to water attack as the Matrix (without CuO). For C8 sample (x = 16 mol%) it was observed a considerable decrease in solubility, which suggests an increased resistance of the vitreous structure to water attack and, therefore, important changes in the structural network. It is known that the biocompatibility of phosphate-based glasses is characterized by a fast resorption kinetics. This is being extensively investigated by other researchers [35,36,37]. 

The most important structural parameter which affects the dissolution of these phosphate glasses is the network connectivity (NC) dependent on the glasses composition [38]. In this case, the NC is critically dependent on composition, especially the changes provoked by the doping of the network modifying cations (Cu ions). Copper ions break the P-O-P bonds (where P is a network former, phosphorus in these glasses) causing the formation of non-bridging oxygens (NBO) and decreasing the network connectivity [39], this being more obvious for x = 16 mol%. At the same time, another parameter that plays an important role in the dissolution of these glasses is the amount and type of Qn phosphate species that can serve as an indicator of the relative amounts of bridging and non-bridging oxygens present in the structure of glass and at the surface.

**Figure 2:**
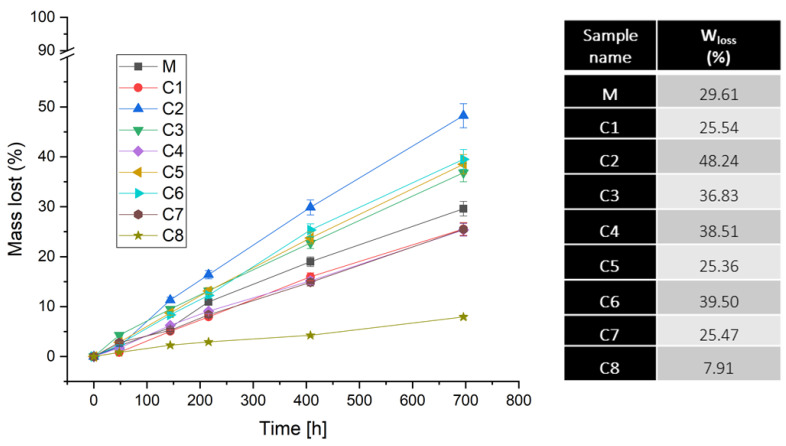
A graph illustrating the weight loss of each piece of glasses over a period of 696 h, respectively 29 days in 10 mL of deionized water.

Figure 2 shows the weight loss of the glass pieces (due to immersion in deionized water) depending on the immersion time. From the first 48 h, the pieces of glasses showed a slight loss of mass, which subsequently increased progressively with the duration of the dive. The most soluble samples were the matrix and the glasses, with up to x = 8 mol% CuO with a mass loss rate between 25.36 and 48.24%. For the C8 sample with the most doping (x = 16 mol%), it was observed a mass loss of only 7.91%, this being the least sensitive to water attack. Despite that, the dissolution rate of the glasses pieces was considerably lower compared to the glasses powder. This can be explained by the simple fact that in the case of glass powder, the particles used have a much higher ratio between the surface area and the volume compared to the pieces of glass; the smaller the size of the glass, the higher the ratio and the higher the reactivity of the contact surface in the implantation medium (deionized water in our experimental design)) [40,41].

#### 3.1.2. pH Measurements

**Figure 3:**
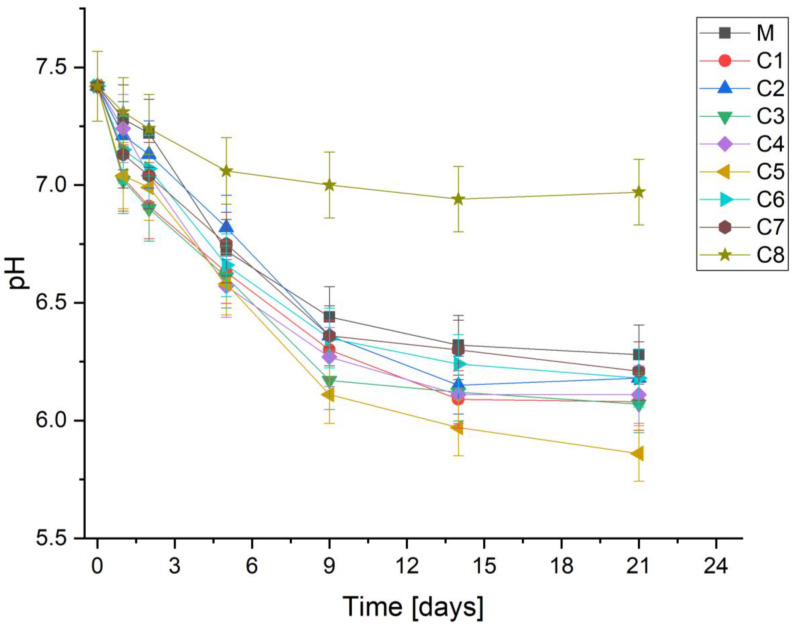
pH variation of PBS without-soaking time for CaF–P_2_O_5_–CaO system containing various CuO.

Figure 3 shows the change in pH of PBS after immersion of the glass samples with the xCuO ∙ (100 − x) [CaF_2_∙3P_2_O_5_∙CaO] glass system where 0 ≤ x ≤ 16 mol% for different time periods. The mechanism of apatite hydroxycarbonate (HCA) formation can be assessed by the pH behavior of the PBS solution after immersion of glasses samples. The pH behavior of all vitreous samples showed similar trends. The pH of the PBS solution after 29 days was decreased significantly from an initial value of 7.42 to 6.28, 6.08, 6.18, 6.07, 6.11, 5.86, 6.18, 6.21, 6.97 for M, C1, C2, C3, C4, C5, C6, C7, and C8, respectively. The lowering of the pH demonstrates the dissolution of the anions on the surface of the glass. Furthermore, the pH of the PBS solution decreased in all samples, which is due to the absorption of calcium, phosphate and fluorine ions from PBS to promote the formation of the HCA layer on the sample surface. The results show that the replacement of P_2_O_5_ with CuO in the present investigation did not significantly alter the bioactivity mechanism of glasses in PBS. However, it is interesting that the C8 (x = 16 mol%) sample reached the highest pH compared to the rest of the samples. This may be due to the high concentration of doping which caused significant changes in the phosphate structure changing the dissolution kinetics due to greater M-O bond strength of copper.

#### 3.1.3. Surface Morphology and EDX

**Figure 4:**
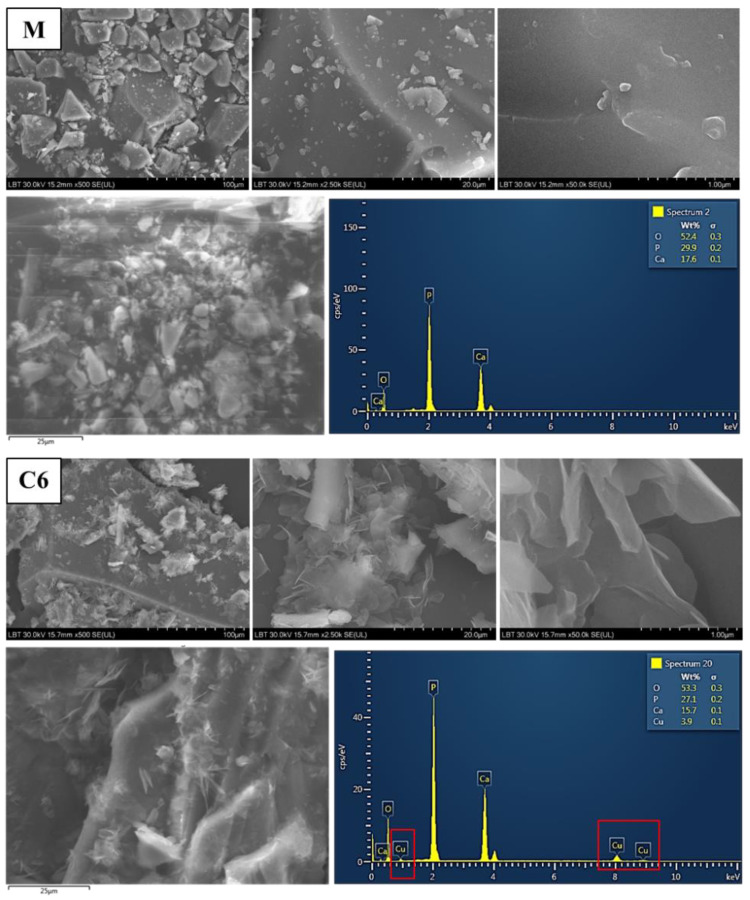
SEM images and EDX spectra for x = 0 and 1 mol% CuO glasses before the dissolution experiments.

The surface morphology of two relevant samples (C6 and M) is shown in Figure 4. Scanning electron microscopy (SEM) images clearly show the various irregular shapes, also revealing particle size. They are angular in shape and have sizes ranging from 20 to 100 µm (Figure 4). The qualitative compositional analysis of the calcium phosphate glasses confirmed the presence of elements P, O, Ca, and Cu in glasses in quantities analogous to the nominal composition of the system. An increase in the emission line of Cu and a decrease in phosphate and calcium lines for glasses C1–C8 was noticed. For samples glass with Cu-dopping ions up to x = 1 mol% (C6), these showed an increase in the emission line of O comparative with the copper-free sample (M). The semi-quantitative EDX analysis provides information about the increase in copper percentage from C1 to C8, parallel with a decrease in percentage of P and O. There were noticed all the characteristic lines of P, O, and Ca for the matrix. The average of the elementary weight percentages was obtained for each sample (Table 3).

#### 3.1.4. FT-IR

This section’s Phosphate-based glass network is based on PO_4_ tetrahedral structural units. As shown in literature [42], because of the sp^3^ hybridization, phosphorus makes four bonds with the oxygen atoms in a tetrahedral configuration. Three of these bonds are equivalent in length (1.55 Å), but the fourth P = O bond is unequivalent and shorter (1.39 Å) than the others, suggesting a π double bond [20,42], inactive from a chemical point of view. As consequence, each tetrahedral structure bridges with other three ones, as the fourth bridge is inactive. When the phosphate-based glass network is doped with different ions deriving from different modifying oxides, the entire glass structure is changed in a direct connection with the content of the modifying oxide.

The Ducel model [43] explains very clearly the way in which phosphate network structural units are changed in the depolymerization mechanism of the network. According to this model there are three types of units in the network structure: (PO_2_)^−^ is the middle unit connected with two phosphorous atoms, PO_3_^2−^ is the ending unit connected to one single phosphorus atom and PO_4_^3−^ is the monomer (isolated) unit, unconnected with any phosphorus atom. During the depolymerization process, there is a continuous transformation of P-O-P bridges from PO_4_ tetrahedral units into PO^2−^, PO_3_^2−^ and then PO_4_^3−^ structural units.

**Figure 5:**
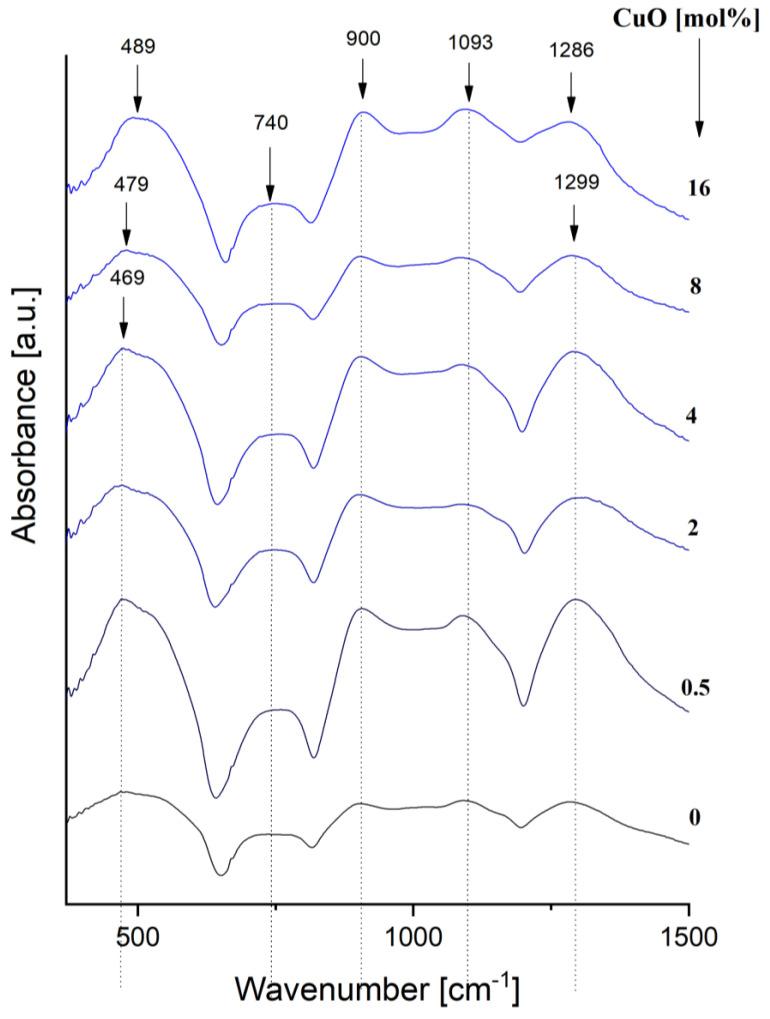
IR spectra of xCuO∙(100 − x) [CaF_2_∙3P_2_O_5_∙CaO].

The present work aims to present the changes induced by the addition of CuO in the xCuO∙(100 − x) [CaF_2_∙3P_2_O_5_∙CaO] glass system with 0 ≤ x ≤ 16 mol%. As is known from the literature [20,42], CuO gradually introduced in the glass network induces a relevant depolymerization of it. However, CuO may also act as a network former in certain amounts and for certain structural configurations [20,44]. For our proposed system, recorded IR spectra (Figure 5) show that CuO plays the role of a modifier agent, inducing the depolymerization of the glass network as the oxide concentration increases. The evolution of the IR bands is consequently influenced by CuO content.

The main IR bands specific for the phosphate network (x = 0) are typical for both middle and ending phosphorous sites (polyphosphate): the band at ~1280–1300 cm^−1^ is attributed to both (PO_2_)^−^ stretching vibrations and to P = O double bond vibration modes; the two bands centered at ~1090 cm^−1^ and ~1180 cm^−1^ are generally attributed to the symmetric stretching of P-O- bonds in PO_4_^3−^ groups and to asymmetric stretching in PO^2−^ groups but also possible to P-O-Ca bond vibrations; the band at ~900 cm^−1^ belongs to P-O-P asymmetric vibrations modes in PO_3_^2−^ units; the two bands at ~720 cm^−1^ and ~780 cm^−1^ are attributed to symmetric stretching of P-O-P bonds in ring units; the band at ~550 cm^−1^ is assigned to O-P-O bending modes in (PO_3_)^2−^ and PO^2−^ units also to Ca-O-P vibration modes; the band centered at ~450 cm^−1^ is attributed to harmonics of bending vibrations [20,45,46,47].

The main bands attributed to the phosphate network suggest a polyphosphate chain of PO_4_ tetrahedra negatively charged and held together through electrostatic forces [20,48,49]. There are two types of oxygen atoms present in the network, BOs bonded to two phosphorous atoms and NBOs connected to one P atom [42,45].

As CuO content increases, some notable changes in the IR band appear. The band at ~1300 cm^−1^ shifts slowly to 1286 cm^−1^. The literature indicates two possible reasons for this shift: a reduced force between P and O as the length of P = O bond increases [20,45] and also to an increase charge of density in the tetrahedral phosphate units reducing the covalent bond and increasing the ionic bond. This makes possible the formation of Cu-O-P bonds (at higher concentration of Cu^2+^ ions) making weaker the initial P = O bond and producing hence the depolymerization of the phosphate network. An important change is related to the reduction of the peak at 1180 cm^−1^ up to its disappearance over 4% CuO content as PO_2_^−^ structures in long P-O-P chains decrease with the depolymerization process. A slightly more intense band at ~1093 cm^−1^ for 16% CuO is also observed as PO_3_^2−^ and PO_4_^3−^ units increase in a number parallel with the formation of more NBOs. The bands in the region 720–740 cm^−1^ and ~900 cm^−1^ are increasing in intensity and this is a clear indication of the fact that the phosphate network is present on the entire considered composition range despite of an increase of P_2_O_5_ content [20,46,47]. The frequent increase of the bands in 720–780 cm^−1^ ranges up to 4% also indicates a change, an increase in the P-O-P bonds in PO_3_^2−^ units. The slow shift and increase in intensity of the band at ~450 cm^−1^ towards 485 cm^−1^ indicate the decrease of the double bond strength when Cu^2+^ connects to the network at their higher concentration. 

#### 3.1.5. EPR

**Figure 6:**
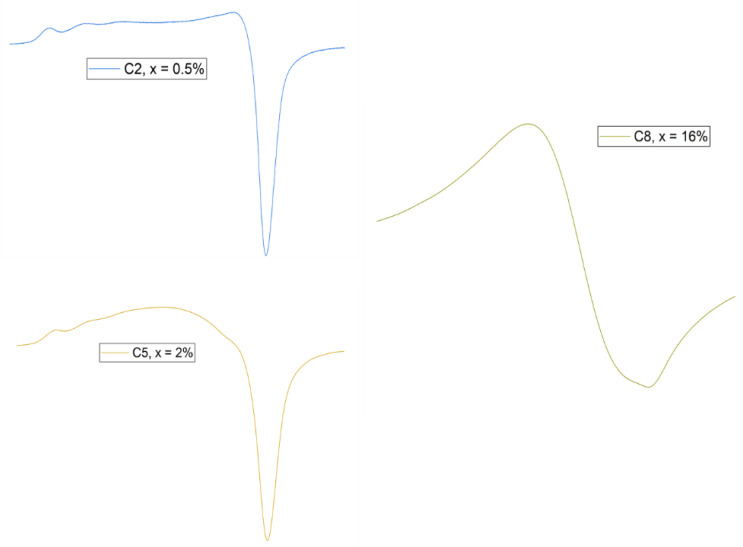
EPR spectra of xCuO∙(100 − x) [CaF_2_∙3P_2_O_5_∙CaO] glass system with x = 0.5, 2 and 16 mol%.

EPR spectra of xCuO∙(100 − x) [CaF_2_∙3P_2_O_5_∙CaO] glass system with 0 ≤ x ≤ 16 mol% are given in Figure 6.

The shape of these spectra up to 4 mol% is typical for Cu^2+^ isolated ions as the quantity of CuO is small in the phosphate network. The parallel absorption band with a quartet of hfs lines is partially well resolved, but the hfs of the perpendicular band is totally unresolved. The increase of the CuO content (over 4 mol%) leads to the formation of a broad resonance line which is attributed in the literature to the clustered Cu^2+^ ions, mainly due to dipole-dipole interactions [13,20,50]. Tensors g⊥, g‖, A‖ and α^2^ coefficient were calculated based on [50] and they are given in Table 4.

All the values calculated for g‖ obey the rule g‖ > g⊥ > ge [51], which indicates a tetragonal elongated octahedron for the coordination of Cu^2+^ ions [20]. On the other hand, unusual large values for g‖ (g‖ > 2.38) obtained for the present system of phosphate glasses (g‖~2.4) and small values for A‖ (A‖ < 120 10 − 4 cm^−1^) especially for a higher content of CuO (for x > 0.75 mol%) were attributed to tetracoordinated Cu^2+^ ions in a tetrahedral local symmetry. In this case, the paramagnetic ”hole” is not seen as a pure 3d orbital, but a mixture of 3d and 4p orbitals [50,52]. A similar tetrahedral coordination was obtained in previous copper-calcium or copper-lead phosphate based glass materials [20]. Moreover, from our experience, the presence of CaF_2_ oxide in the glass matrix increases the density of the electronic cloud around the paramagnetic ion reducing the tetragonal character of the coordination. According to different opinions [20,51] this fact leads to a higher value for g‖ and a lower one for A‖. Thus, as evaluated from the spectra, EPR parameters indicate a very high degree of sensitivity to the glass composition materialized in the changing of the local environment of copper ions.

The very small variation calculated for the α parameter related to the σ-bond between Cu^2+^ ion and its ligands (α^2^ = 0.8–0.84) indicates a very low covalency degree for the entire concentration range in the present glass system [50]. This is also consistent with our IR results that indicated a depolymerization of the phosphate network especially for x > 4 mol%, when the charge density on PO_4_ tetrahedral groups is increased, and in consequence a more ionic and a less covalent bonding is produced [50,52,53]. The presence of Cu^2+^ clustered ions in the phosphate network for x > 8 mol% is also consistent with its modifier role.

**Figure 7:**
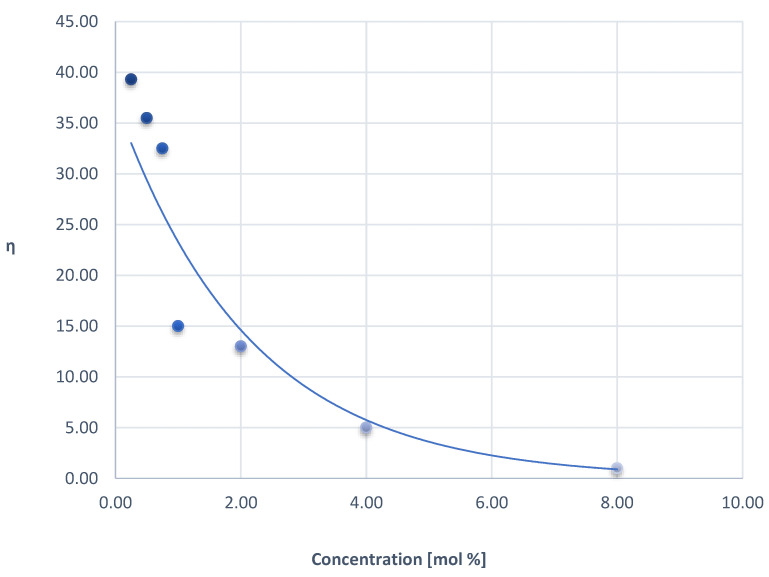
EPR concentration dependence of asymmetry parameter.

By defining the asymmetry parameter η as the ratio of g ⊥ and g‖ absorptions heights, a decrease of η value is observed when increasing the number of Cu^2+^ ions (Figure 7). At x ≥ 8 mol% the hfs disappears and the EPR spectra takes an isotropic form, a symmetric broad line because of the exchange interactions between the paramagnetic Cu^2+^ ions [20,50,53] copper.

### 3.2. In Vitro Biological Evaluation

#### Evaluation of Cytotoxicity-MTT Assay

In addition to structural characterization, and functional and chemical characterization, cytotoxicity analysis is also very important in evaluating the antitumor character of bioactive glass samples.

The MTT assay was performed to investigate the in vitro cytotoxicity of the samples after 24 h in which the samples were exposed to tumor epithelial A375 cells, and the cell/extract of bioglass C6/M interactions, such as differentiation and proliferation were studied.

**Figure 8:**
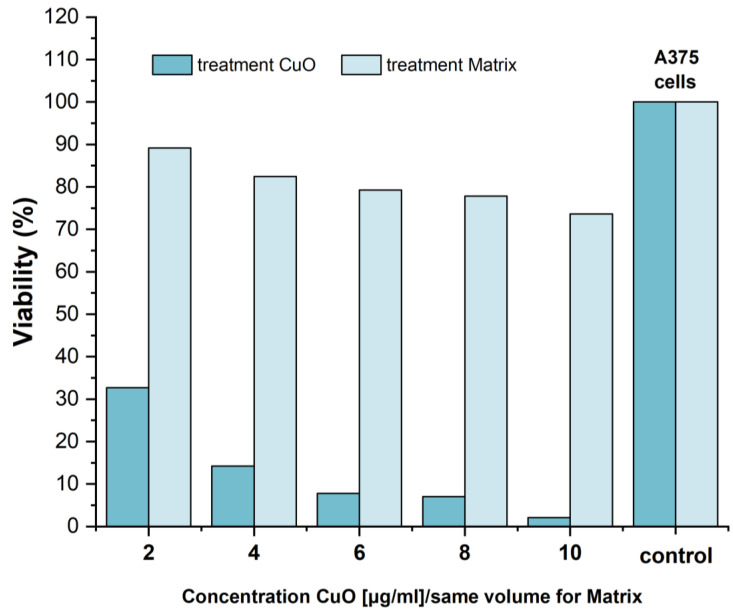
MTT cytotoxicity assay results using 10-day incubation liquid extracts from C6 glass in different concentrations; *n* = 5.

Usually, cytotoxicity is associated with the reactivity of compounds [31]. If ionic exchange is more intense between glass particles and the environment, then the surface modification leads to a sample’s higher toxicity. The cytotoxicity of melt-derived glass samples with and without CuO content onto human melanoma cells is shown in Figure 8. In the case of extract of glass C6 (x = 1 mol%), an aggressive reduction in cell viability was observed for all the concentrations (2, 4, 6, 8, and 10 μg/mL). The results show a linear relationship between the number of cells and the concentration of CuO ions, this treatment being very toxic against the tumor cells. The extract of the matrix provides significant survival cell numbers compared with that of C6 extract, thus demonstrating that the treatment without copper had good biocompatibility on the tumor cells. The maximum viability was attained at an equivalent volume of 2 μg/mL CuO and decreases as the equivalent volume of CuO increases. It should be noted that the Cu-based treatment induced a powerful antiproliferative effect on A375 tumor cells. The inferred mechanism behind this cytotoxic activity can be associated with both blockage of cellular oxidative defenses and apoptosis through the generation of reactive oxygen species (ROS) [54,55,56,57,58].

## 4. Conclusions

xCuO∙(100 − x) [CaF_2_∙3P_2_O_5_∙CaO] glass system was obtained within the 0 ≤ x ≤ 16 mol% composition range by the conventional melt-quenching method. In order to characterize this system, all the samples obtained were analyzed by dissolution tests, pH measurements, FTIR, SEM-EDX, EPR and MTT analyzes.

Solubility tests showed that the dissolution rate of glasses is grown for all samples in the aqueous medium, except for the C8 sample (x = 16 mol%). In this case, the network connectivity was significantly affected by the increased amount of CuO. Moreover, pH measurements revealed low values for all samples earlier than 48 h.

SEM analysis illustrated that the structure and the homogeneity of copper containing calcium fluorine phosphate glasses does not considerably change with doping concentration. EDX analysis confirmed the presence of all elements (P, O, Ca and Cu) according to the batch compositions.

FT-IR spectra highlighted the contribution of copper oxide on the glass structure and it plays the network modifier role determining the formation of NBOs. EPR results indicated a very high sensitivity of Cu^2+^ to the local environment, especially for x ≥ 4 mol%.

An MTT test was carried out only for the glass sample with x = 1 mol% CuO and standard (without CuO). It showed that CuO-based treatment had an antiproliferative potential against the A375 tumor cell line, while the treatment without CuO presented good biocompatibility with over 70% viability. The cytotoxicity of CuO ions gave a promising outcome which makes them an alternative anticancer support for skin cancer cells.

## Figures and Tables

**Table 1:** Chemical compositions (wt%) of CaO-P2O5-CaF:CuO glasses.

Sample Glass	P_2_O_5_	CaF_2_	CaO	CuO
M	76	13.9	10	0
C1	75.9	13.9	10	0.2
C2	75.8	13.9	10	0.4
C3	75.6	13.9	10	0.5
C4	75.5	13.8	9.9	0.7
C5	75	13.7	9.9	1.4
C6	73.9	13.5	9.7	2.9
C7	71.6	13.1	9.4	5.8
C8	67	12.3	8.8	11.9

**Table 2:** The reagents used in the preparation of the buffer solution (PBS, pH 7.4).

Reagent Chemicals (Chempur)	Amount (mg)	Concentration (M)
NaCl (mw: 58.4 g/mol)	8000	0.137
KCl (mw: 74.551 g/mol)	200	0.0027
Na_2_HPO_4_ (mw: 141.96 g/mol)	1440	0.01
KH_2_PO_4_ (mw: 136.086 g/mol)	245	0.0018

**Table 3:** The average overall chemical composition of xCuO∙(100 − x) [CaF_2_∙3P_2_O_5_∙CaO] glass system by EDX analysis.

	Element Wt (%)			
Sample Glass	O	P	Ca	Cu
M	52.40	29.90	17.60	—
C1	59.35 ± 2.0	26.20 ± 0.7	14.12 ± 1.3	0.30 ± 0.1
C2	56.90 ± 1.5	27.40 ± 0.9	15.15 ± 0.7	0.60 ± 0.1
C3	57.40 ± 0.5	26.00 ± 0.2	15.90 ± 0.6	0.80 ± 0.2
C4	62.25 ± 2.8	23.95 ± 1.4	12.75 ± 1.2	1.05 ± 0.3
C5	61.00 ± 2.8	24.80 ± 1.4	13.10 ± 1.2	1.10 ± 0.3
C6	55.20 ± 1.9	26.55 ± 0.6	14.90 ± 0.8	3.35 ± 0.6
C7	50.65 ± 1.9	28.95 ± 0.4	15.6 ± 1.2	5.00 ± 0.5
C8	45.20	24.50	15.70	14.60

**Table 4:** EPR parameters for xCuO∙(100 − x) [CaF_2_∙3P_2_O_5_∙CaO].

x mol%	g_‖_	g_⊥_	A_‖_ (10^−4^ cm^−1^)	α^2^
0.25	2.42	2.03	136	0.81
0.5	2.41	2.04	159	0.80
0.75	2.44	2.04	119	0.84
1	2.44	2.03	109	0.84
2	2.42	2.03	108	0.81
4	2.43	2.03	106	0.81

## Data Availability

Not applicable.
